# Supplementary material for: Wnt/β-catenin signaling is an evolutionarily conserved determinant of chordate dorsal organizer
Source: eLife. 2020 May 26;9:e56817. doi: 10.7554/eLife.56817 (PMC7292647; doi:10.7554/eLife.56817)
Supplement: Supplementary file 1. [file elife-56817-supp1.docx]

**Supplementary table 1**

Chemicals used in this study to affect the signaling pathways

| **CHIR99021** | Inhibits both glycogen synthase kinase3ß (GSK3β) and GSK3α that leads to the stabilization of ß-catenin and activation of Wnt/ß-catenin signalling (109, 110) |
| --- | --- |
| **C59** | Inhibits a key enzyme in Wnt biosynthesis, the membrane bound O-acyltransferase porcupine (PORCN), which is essential for the O-palmitoylation of Wnts required for Wnt secretion and biological activity (109, 111) |
| **SB 505124** | Selectively and concentration-dependently inhibits ALK4-, ALK5-, and ALK7-dependent activation of downstream cytoplasmic signal transducers, Smad2 and Smad3 (112) |
