# Supplementary material for: Wnt/β-catenin signaling is an evolutionarily conserved determinant of chordate dorsal organizer
Source: eLife. 2020 May 26;9:e56817. doi: 10.7554/eLife.56817 (PMC7292647; doi:10.7554/eLife.56817)
Supplement: Transparent reporting form [file elife-56817-transrepform.docx]

***eLife’s* transparent reporting form**

We encourage authors to provide detailed information *within their submission* to facilitate the interpretation and replication of experiments. Authors can upload supporting documentation to indicate the use of appropriate reporting guidelines for health-related research (see [EQUATOR Network](http://www.equator-network.org/%20)), life science research (see the [BioSharing Information Resource](https://biosharing.org/)), or the [ARRIVE guidelines](http://www.plosbiology.org/article/info:doi/10.1371/journal.pbio.1000412) for reporting work involving animal research. Where applicable, authors should refer to any relevant reporting standards documents in this form.

If you have any questions, please consult our Journal Policies and/or contact us: [editorial@elifesciences.org](mailto:editorial@elifesciences.org).

**Sample-size estimation**

- You should state whether an appropriate sample size was computed when the study was being designed
- You should state the statistical method of sample size computation and any required assumptions
- If no explicit power analysis was used, you should describe how you decided what sample (replicate) size (number) to use

Please outline where this information can be found within the submission (e.g., sections or figure legends), or explain why this information doesn’t apply to your submission:

Sample size was not explicitely established prior to our experiments.

Experiments performed on the batches of the embryos (Figure 4, Figure 5, Figure6, Figure 7, Figure 8, Figure 9, Figure7-figure supplement 1) were designed to include as many batches as spawning conditions allowed, but test no less than 3 batches, and perform the experiments across different spawning days.

Experiments using qualitative techniques such as whole-mount *in situ* hybridization, immunofluorescence and confocal microscopy imaging (Figure 1, Figure 2, Figure 3, Figure1 -figure supplement 1, Figure2-figure supplement 1) were designed to produce internally consistent data to allow the assessment of general patterns.

**Replicates**

- You should report how often each experiment was performed
- You should include a definition of biological versus technical replication
- The data obtained should be provided and sufficient information should be provided to indicate the number of independent biological and/or technical replicates
- If you encountered any outliers, you should describe how these were handled
- Criteria for exclusion/inclusion of data should be clearly stated
- High-throughput sequence data should be uploaded before submission, with a private link for reviewers provided (these are available from both GEO and ArrayExpress)

Please outline where this information can be found within the submission (e.g., sections or figure legends), or explain why this information doesn’t apply to your submission:

Pharmacological treatment experiments: the treatment on the individual batch from different females was defined as a biological replicate.

Microinjection experiments: microinjection of the eggs from individual batches was defined as a biological replicate.

Luciferase assay: each experiment performed in triplicate assays was defined as biological replicate.

Each treatment, microinjection and luciferase assay was performed at least three times.

In methods it is described in the section *In situ* hybridization and immunostaining:

‘In-situ hybridization was performed as previously described (38) and carried out at least three times with 20 embryos each time for each gene and experimental conditions.’

‘Each individual staining of protein was performed with at least 20 embryos repeatedly at least three times.’

For luciferase assay it is referred to Kozmikova et.al.

The numbers of embryos are indicated in the right bottom corner of the panel.

**Statistical reporting**

- Statistical analysis methods should be described and justified
- Raw data should be presented in figures whenever informative to do so (typically when N per group is less than 10)
- For each experiment, you should identify the statistical tests used, exact values of N, definitions of center, methods of multiple test correction, and dispersion and precision measures (e.g., mean, median, SD, SEM, confidence intervals; and, for the major substantive results, a measure of effect size (e.g., Pearson's r, Cohen's d)
- Report exact p-values wherever possible alongside the summary statistics and 95% confidence intervals. These should be reported for all key questions and not only when the p-value is less than 0.05.

Please outline where this information can be found within the submission (e.g., sections or figure legends), or explain why this information doesn’t apply to your submission:

Statistical analysis was done for experiment shown in Figure 6Bc-Bd. Statistical significance was determined using Student t-test in Microsoft Excel. Graph values represent the average of 10 embryos of each group. P-value was 6.60433E-05.

Statistical analysis was done for experiment shown in Figure 9-figure supplement 1. Statistical significance was determined using Student t-test in Microsoft Excel. P-value is indicated in the legend of Figure 9-figure supplement 1.

(For large datasets, or papers with a very large number of statistical tests, you may upload a single table file with tests, Ns, etc., with reference to sections in the manuscript.)

**Group allocation**

- Indicate how samples were allocated into experimental groups (in the case of clinical studies, please specify allocation to treatment method); if randomization was used, please also state if restricted randomization was applied
- Indicate if masking was used during group allocation, data collection and/or data analysis

Please outline where this information can be found within the submission (e.g., sections or figure legends), or explain why this information doesn’t apply to your submission:

For pharmacological treatments, embryos were taken from the same spawning batches. No selection was done to allocate embryos into either control or experimentally manipulated groups. No embryo was excluded from the experiment from either control or experimentally manipulated group.

**Additional data files (“source data”)**

- We encourage you to upload relevant additional data files, such as numerical data that are represented as a graph in a figure, or as a summary table
- Where provided, these should be in the most useful format, and they can be uploaded as “Source data” files linked to a main figure or table
- Include model definition files including the full list of parameters used
- Include code used for data analysis (e.g., R, MatLab)
- Avoid stating that data files are “available upon request”

Please indicate the figures or tables for which source data files have been provided:

Does not apply.
